# Mas-Related G Protein-Coupled Receptor-X2 (MRGPRX2) in Drug Hypersensitivity Reactions

**DOI:** 10.3389/fimmu.2018.03027

**Published:** 2018-12-20

**Authors:** Grzegorz Porebski, Kamila Kwiecien, Magdalena Pawica, Mateusz Kwitniewski

**Affiliations:** ^1^Department of Clinical and Environmental Allergology, Jagiellonian University Medical College, Krakow, Poland; ^2^Department of Immunology, Faculty of Biochemistry, Biophysics and Biotechnology, Jagiellonian University, Krakow, Poland

**Keywords:** anaphylaxis, drug allergy, drug hypersensitivity, mast cells, MRGPRX2

## Abstract

The human ortholog MRGPRX2 and the mice ortholog, Mrgprb2 are activated by basic secretagogues and neurokinins. A number of commonly used small-molecule drugs (e.g., neuromuscular blocking agents, fluoroquinolones, vancomycin) have been recently shown to activate these receptors under *in vitro* experimental conditions, what results in mast cell degranulation. The above drugs are also known to cause IgE-mediated anaphylactic reactions in allergic patients. The new findings on mechanisms of drug-induced mast cell degranulation may modify the current management of drug hypersensitivity reactions. Clinical interpretation of mild drug-provoked hypersensitivity reactions, interpretation of skin test with a drug of interest or further recommendations for patients suspected of drug allergy are likely to be reconsidered. In the paper we discussed future directions in research on identification and differentiation of MRGPRX2-mediated and IgE-dependent mast cell degranulation in patients presenting clinical features of drug-induced hypersensitivity reactions.

## Anaphylaxis

Anaphylaxis is a well-recognized, life-threatening medical condition. The clinical definition of anaphylaxis involves the observation of acute symptoms in two or more body systems or is associated with upper respiratory complications leading to asphyxiation and/or hypotension, which may result in cardiovascular collapse. Pruritus, urticaria, angioedema, bronchospasm and wheezing, hypotension, nausea, vomiting, and diarrhea are the usual clinical manifestations (1, 2). Because of this plethora of symptoms, anaphylaxis continues to be under recognized in 80% of the patients, who are admitted to the emergency departments of hospitals after medical procedures associated with exposure to drugs (3).

An anaphylactic reaction may occur via an IgE-mediated (allergic) or a non-IgE-dependent mechanism (previously called a pseudo-allergic or anaphylactoid mechanism) (4, 5). From a clinical point of view they are impossible to distinguish by standard investigation. The effector phase of reaction responsible for the above clinical picture is caused by the release of mediators from mast cells and basophils. Mediators comprise histamine, which is well-known and has been extensively studied for years; platelet-activating factor (PAF), which is relevant as a decrease in its degradation predisposes to severe anaphylaxis (6) and many others, such as neutral proteases (tryptase, chymase, carboxypeptidase), proteoglycans (heparin, chondroitin sulfate), chemoattractans, and products of arachidonic acid metabolism. Possible triggers for anaphylaxis include food and environmental allergens such as nuts, egg, seafood, latex, Hymenoptera venoms (e.g., bee, wasp), as well as drugs, which are described in more detail below (3).

## Drugs as Triggers for Anaphylactic Reactions

In parallel to the increase in the use of pharmacotherapy, drug hypersensitivity reactions (DHR), including anaphylaxis have increased significantly worldwide in the last 20 years (3). Some groups of drugs have been well-known as being triggers of anaphylactic reactions for years, for example beta-lactam antibiotics or classical chemotherapy agents (carboplatin, cisplatin, taxanes) (7). Other drugs, such as monoclonal antibodies (mAbs), have entered into clinical practice more recently, but they may also provoke immediate hypersensitivity reactions. Chimeric mAbs (rituximab, infliximab) are reported to induce these reactions more often than humanized mAbs, however even fully human mAbs differ in glycosylation patterns, which may result in the formation of allergenic determinants and subsequent anaphylactic reactions (8).

Some drugs may elicit both IgE-mediated and non-IgE-mediated hypersensitivity reactions. Neuromuscular blocking agents (NMBAs) are considered to induce a majority of the anaphylactic reactions which may occur during general anesthesia (9, 10). Interestingly, up to 85% of these reactions occur in patients without previous exposure to NMBAs (11). Drug-induced anaphylactic reaction in drug-naïve patients can be explained by IgE cross-sensitization or the presence of a non-IgE-dependent mechanism. Fluoroquinolones represent another group of drugs which may cause anaphylactic reactions in drug-naïve patients (12, 13). Therefore, it is unlikely that these reactions are the result of drug-specific IgE linking to FcεRI. An alternative mechanism, based on mast cells activation by means of the G-protein-coupled receptor X2 (MRGPRX2) (14), will be discussed later. Patients with immediate reaction to fluoroquinolones frequently do not demonstrate IgE reactivity (15, 16) and again positive skin tests may be the result of non-specific histamine release, especially when observed in individuals tolerant to fluoroquinolones (17, 18). Similarly, vancomycin, a glycopeptide antibiotic, is known to trigger not only typical IgE-mediated immediate reactions, but also the so called “red man syndrome.” This clinical entity is associated with erythema and itchy rashes involving the face, neck, and upper torso (19). How vancomycin provokes the mast cell degranulation responsible for symptoms of “red man syndrome” has not been sufficiently understood so far.

Having the above in mind, one should also take into account that many drugs may cause IgE-mediated anaphylaxis without previous exposure, because of cross-reactivity between specific structures that are found in different molecules (e.g., substituted ammonium groups on quinolones and NMBAs). Moreover, IgE response may occur to simple chemicals after coupling them to carrier proteins, to polyamines by recognition of primary amine groups, as well as to a number of unreactive drugs having neither suitable functional groups nor properties to form drug-carries antigens (20). However, a huge number of drugs have been shown to induce IgE-mediated reactions, laboratory investigations aimed at identification of responsible factor are relatively poorly available and/or existing routine diagnostic methods have low sensitivity. Therefore, the fact that IgE to drugs is not found does not rule out its presence.

## Mast Cells

Mast cells (MC) have largely been considered to possess a key role in the production of immediate allergic reactions, as upon activation they release a variety of mediators from stored granules. But mast cells also participate in homeostasis and inflammation, innate and adaptive immunity, as well as angiogenesis in a variety of tissues (21). Thus, they are mostly found in the host-environment interface, such as the skin, lung, or gastrointestinal tract, which are challenged by a variety of extrinsic agents—allergens and pathogens. During the maturation process MC develop differences in granule composition and tissue-specific receptor patterns (22, 23). Mucosal (MCT) and connective tissue (MCTC) mast cells represent two classical subtypes of these cells.

**Figure 1:**
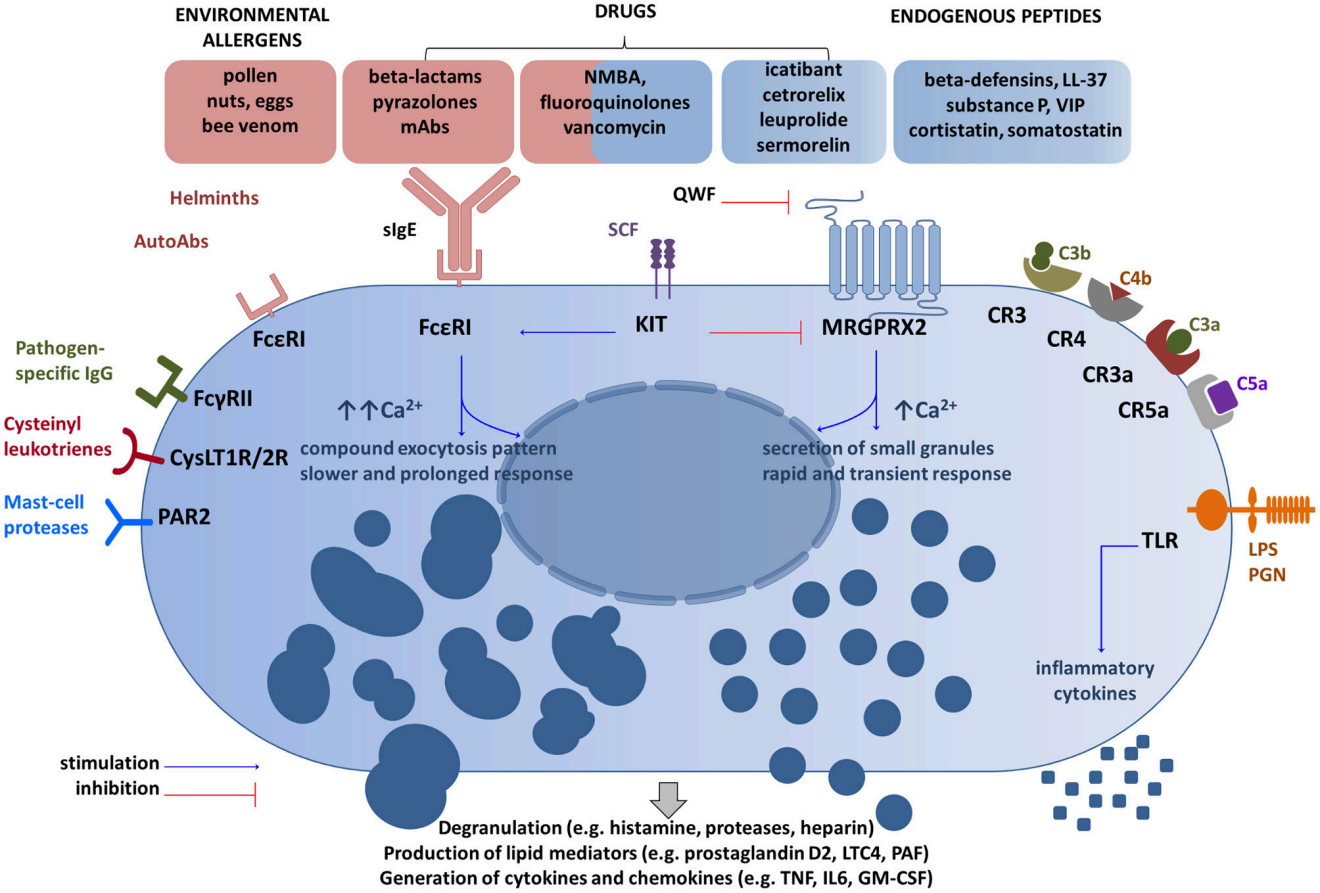
Main receptor systems and examples of ligands involved in mast cell activation. Drugs can activate mast cells through both sIgE-dependent mechanism and the MRGPRX2 receptor (14, 26, 38). These activation routes are independent and inversely regulated by SCF (51). QWF inhibits activation of human MRGPRX2 by a number of basic secretagogues and medications (47, 50, 52), but not by LL-37 (53). Granule processing and response program after the MRGPRX2 engagement differ from FcεRI-mediated response (32, 33). Several other representative MC receptor systems and ligands are shown (28, 29). CR, complement receptor for corresponding complement components; CysLT1R/2R receptors, cysteinyl leukotrienes; FcγRII, a low-affinity receptor for IgG; FcεRI, the high affinity IgE receptor; GM-CSF, granulocyte/macrophage colony-stimulating factor; IL, interleukin; KIT, mast/stem cell growth factor receptor (CD117); LPS, lipopolysaccharide; LTC4, leukotriene C4; Abs, antibodies; MRGPRX2, Mas-Related G Protein-Coupled Receptor-X2; NMBA, neuromuscular blocking agents; PAR2, protease-activated receptor 2; PGN, peptidoglycan; SCF, stem cell factor; sIgE, specific IgE; TLR, Toll-like receptor; TNF, tumor-necrosis factor; QWF–tripeptide (the glutaminyl-D-tryptophylphenylalanine); VIP, vasoactive intestinal peptide. The schematic drawings were generated by modifying images obtained from *Motifolio* (Motifolio Inc., Elliocott City, MD, USA).

Allergens, (glyco) proteins, or auto-antibodies directed against the FcεRI receptor (FcεRI) or receptor-bound IgE antibodies cause MC degranulation after cross-linking and aggregation of the surface-bound FcεRI (24, 25). However, mast cell degranulation can be also achieved by non-IgE-dependent pathways thanks to a wide range of surface receptors, including toll-like receptors (TLR), protease-activated receptors (PARs), or Mas-related G-protein coupled receptor member X2 (MRGPRX2) (14, 26–29). Therefore, MCs are able to identify and respond to a number of various exogenous (e.g., pathogen-associated molecular patterns, some contents of insect venom, many drugs, polycationic molecules such as compound 48/80) and endogenous stimuli provoking degranulation (e.g., cytokines, anaphylatoxins, chemokines, IgG, neuropeptides such as substance P) (25, 26, 30–32). Various stimuli are processed in different ways and may result in distinct degranulation programs as demonstrated by Gaudenzio et al. (33) (Figure 1). Therefore, MC activation is not a uniform event. Some clinical entities such as systemic mastocytosis and chronic urticaria show that a number of co-factors can exacerbate symptoms by triggering mast cells (e.g., physical factors such as heat and cold, pressure, non-steroidal anti-inflammatory drugs, or iodinated contrast media) (4, 5, 34–36).

Upon activation, MCs release a myriad of mediators responsible for creating a clinical picture of immediate reaction. Some of these mediators are preformed (histamine, proteases, heparin), whereas others are newly formed (e.g., thromboxane, prostaglandin D2, leukotriene C4, tumor necrosis factor alpha) (37).

## Mas-related G-protein Coupled Receptor X2 (MRGPRX2)

Systemic mast cell degranulation responsible for anaphylaxis is in many instances IgE-mediated, but in a substantial number of MC degranulation occurs without IgE involvement (26). Of great interest in this regard is a landmark study by McNeil et al. (14), who investigated the Mastocyte-related G-protein coupled receptor X2 (MRGPRX2), and its ortholog, the mouse protein-coupled receptor, Mas-related gene (Mrg) receptor Mrgprb2. They were able to demonstrate that this receptor upon activation with some drugs is capable of producing direct MC degranulation and anaphylactic reactions. MRGPRX2, a member of the Mas-related gene family, was found to be expressed in sensory neurons, mast cells and, most recently, in keratinocytes (38–41). MRGPRX2 mRNA is present in adipose tissue, esophagus, urinary bladder, lungs with the highest levels found in skin (Supplementary Figure S1A). Transcripts were not detected in kidney, liver, ovary, or pancreas (42–44). MRGPRX2 and Mrgprb2 mRNA was detected in adipose tissue (43, 45). Our results show that Mrgprb2 is present in visceral and subcutaneous mouse fat pads but not in sorted, mature adipocytes (Supplementary Figure S1B). Thus, adipose tissue resident cells seem to be a source of Mrgprb2 mRNA but not adipocytes. We have also confirmed that Mrgprb2 transcripts are present in the skin. Surprisingly, we were unable to detect Mrgprb2 mRNA in the urinary bladder, a result that is inconsistent with available data (45). These discrepancies can be attributed to differences in the immune state of the animals, divergences in RNA-seq databases, differences in primers specificity and the like.

Skin seems to be an important organ associated with MRGPRX2-dependent reactions. Beside keratinocytes, the MRGPRX2 is expressed at high levels in MC_TC_ in the skin. The increase in the absolute number and in percentage of MrgX2+ MC in all MC was observed in the skin of patients with chronic urticaria (46). Human beta-defensins secreted by keratinocytes were shown to induce degranulation in human mast cells via MRGPRX2 (47). Taking the above into consideration interactions between keratinocytes and mast cells may therefore be of potential interest.

Downstream signaling from MRGPRX2 involves activation of the phospholipase C-beta pathway, which ends in the release of the preformed and the *de novo* synthesized mediators (26, 48). MRGPRX2-mediated responses seem to be more rapid, but transient in comparison to IgE-triggered events (33, 48). The canonical secretagogues activating MRGPRX2 include basic peptides (substance P, VIP, cortistatin, somatostatin), proadrenomedullin amino-terminal 20 peptide, fragment 9–20 [PAMP(9–20)], some drugs (e.g., morphine, hydrocodone) and cationic proteins capable of producing direct MC degranulation (46, 49–51). McNeil et al. (14) explored also the cationic peptidergic drugs, which are given subcutaneously or intramuscularly and frequently induce injection-site reaction: a local edema accompanied by itch and erythema (Supplementary Figure S2). They showed that representatives of common commercially available drugs (e.g., icatibant, cetrorelix, leuprolide, sermorelin) activate mast cells in an MRGPRX2-dependent manner. Next, they focused on intravenous drugs representing small molecules containing a tetrahydroisoquinoline (THIQ) motif or similar structure, namely neuromuscular blocking agents (NMBAs) and fluoroquinolones. They found that NMBAs except succinylcholine and the four fluoroquinolones approved for intravenous use (ciprofloxacin, moxifloxacin, levofloxacin, ofloxacin) activate MCs through MRGPRX2 and Mrgprb2 (14).

Of note is that the loss of the receptor in Mrgprb2-null mutant mice did not alter the IgE-mediated reaction, but abolish secretagogue-induced histamine release (14). In another set of experiments, the tripeptide abbreviated as QWF (glutaminyl-D-tryptophylphenylalanine) was shown to inhibit activation of human MRGPRX2 by basic secretagogues, such as compound 48/80, and also by atracurium (NMBA) and ciprofloxacin (52). The same research group demonstrated that the aforementioned vancomycin activates human MRGPRX2 and that such activation is prevented by QWF (Figure 1). Moreover, predictive molecular modeling experiments have confirmed that a single amino acid residue mutation in MRGPRX2 (Glu164Arg) prevents the interaction between the receptor and common secretagogues (substance P and compound 48/80). On the other hand, another receptor agonist—LL-37, an antimicrobial peptide, activates both native receptors and mutant receptors, which indicates the presence of distinct activation sites for some receptor agonists (53). In a very recent study, Navines-Ferrer et al. (54) tested several drugs used in perioperative procedures and anesthesia including opiates (morphine, remifentanil), NMBAs (cisatracurium, rocuronium), iodinated contrast agents (meglumine amidotrizoate, iohexol, iomeprol), antibiotics (vancomycin, teicoplanin, amoxicillin-clavulanic acid), nonsteroidal anti-inflammatory drugs (NSAIDs; diclofenac), and anesthetics (propofol). Among those listed cisatracurium, morphine, vancomycin, meglumine amidotrizoate, and iomeprol induced LAD2 mast cells degranulation mediated by MRGPRX2. However, the doses of meglumine amidotrizoate and iomeprol exceeded the concentration usually administrated to patients. Moreover, it was demonstrated that sera collected from patients who had experienced anaphylactoid reaction during anesthesia induced MC degranulation via MRGPRX2 dependent manner.

Single nucleotide polymorphisms (SNPs) are among the most common types of genetic variations. Missense SNPs are thought to affect the structure, interactions and properties of proteins (55, 56). SNPs may be linked with MRGPRX2 variants that might predispose individuals to hyperactivation by changing the structure of MRGPRX2. Yang and colleagues (57) found three specific amino acid substitutions in MRGPRX2 protein: Asn16His, Asn62Ser, and Phe78Leu. Our analyses of SNP NCBI databases and literature review (58) revealed 30 SNPs within coding regions of the human MRGPRX2 locus (Table 1). Two of the most common SNPs with a minor allele frequency (MAF) of 0.3185 and 0.1130 result in amino acid substitution from asparagine to tryptophan at 62 position (Asn62Thr) and asparagine to histidine at 16 position (Asn16His), respectively. It is consistent with the results published by Yang et al. (57). The Asn62Thr affects the cytoplasmic domain 1 (CPD1) of MRGPRX2 and Asn16His extracellular domain 1 (ECD1) as predicted by Phobius (53). Other SNPs are rare or very rare (MAF < 0.01). We have not found any SNP substituting amino acid at 164 position that was shown to change MRGPRX2 binding of substance P and compound 48/80 *in vitro* (53). Very rare SNP with MAF of 0.0014 affects the predicted by Reddy et al. (53) MRGPRX2 binding pocket at 243 position (Trp243Arg) located within transmembrane domain 6 (TMD6). However, the extracellular domains of GPCRs are usually involved in ligand/receptor recognition (57). Among all detected missense SNPs only five may affect extracellular domains: Pro6Thr, Asn16His, Gly165Glu, Asp252Tyr, His259Tyr. However, the most recent study by Alkanfari et al. (58) showed that RBL-2H3 cells expressing one of four missense SNPs (Gly165Glu, Asp184His, Trp243Arg, His259Tyr; Table 1) failed to respond to MRGPRX2 ligands including substance P, hemokinin-1, human β-defensin-3, and icatibant. This may have important clinical implications. Patients harboring listed SNPs may be protected from drug-induced mast cell degranulation and hypersensitivity reactions. Further studies are needed to determine how SNPs may change the ligand binding properties of MRGPRX2.

**Table 1:** The list of SNPs within coding regions of the human MRGPRX2 locus.

	**SNP ID**	**Mutation**	**Nucleotide variation**	**MAF**	**Amino acid change**	**MRGPRX2 domain**	**Response to ligands gained/lost/not changed**
1.	rs10833049	Missense	T > G	C = 0.3185	Asn62Thr	CPD1	n.d.
			T > C		Asn62Ser		
2.	rs11024970	Missense	T > G	G = 0.1130	Asn16His	ECD1	Not changed (HK1, SP, IC, hBD3) (58)
3.	rs11823569	Missense	C > T	T = 0.0066	Val43lle	TMD1	Not changed (HK1, SP, IC, hBD3) (58)
4.	rs564668393	Missense	A > G	G = 0.0066	Ser284Pro	CPD4	n.d.
5.	rs79763999	Missense	A > G	G = 0.0032	Phe78Leu	TMD2	Not changed (HK1, SP, IC, hBD3) (58)
6.	rs60756581	Missense	G > A	A = 0.0022	Arg140Cys	CPD2	n.d.
			G > C		Arg140Gly		
7.	rs114017828	Missense	T > A	C = 0.0022	Met324Leu	CPD4	n.d.
			T > C		Met324Va		
8.	rs145992601	Missense	G > C	C = 0.0020	Leu31Val	TMD1	Not changed (HK1, SP, IC, hBD3) (58)
9.	rs117328742	Missense	A > C	C = 0.0016	Ser313Arg	CPD4	n.d.
10.	rs150365137	Missense	A > G	G = 0.0014	Trp243Arg	TMD6	lost (HK1, SP, IC, hBD3) (58)
11.	rs75443524	Missense	T > A	C = 0.0010	Arg61Trp	CPD1	n.d.
			T > C		Arg61Gly		
12.	rs572320540	Missense	C > A	A = 0.0010	Ala74Ser	TMD2	n.d.
			C > T		Ala74Thr		
13.	rs118176470	Missense	A > G	G = 0.0006	Val108Ala	TMD3	n.d.
14.	rs140862085	Missense	G > A	A = 0.0004	His259Tyr	ECD4	Lost (HK1, SP, IC, hBD3) (58)
15.	rs542994968	Nonsense	C > T	T = 0.0004	Trp190Ter	TMD5	n.d.
16.	rs572101439	Missense	T > C	C = 0.0002	Thr224Ala	TMD6	n.d.
17.	rs564709381	Missense	G > T	T = 0.0002	Pro6Thr	ECD1	n.d.
18.	rs550191582	Missense	T > C	C = 0.0002	Ser103Gly	TMD3	n.d.
19.	rs543158275	Missense	C > G	G = 0.0002	Val51Leu	TMD1	n.d.
20.	rs531328060	Missense	C > T	T = 0.0002	Met196lle	TMD5	n.d.
21.	rs530355228	Missense	G > C	C = 0.0002	Pro322Ala	CPD4	n.d.
22.	rs372986472	Missense	C > A	A = 0.0002	Asp252Tyr	ECD4	n.d.
23.	rs201846837	Missense	C > G	G = 0.0002	Met119lle	TMD3	n.d.
24.	rs201177657	Missense	G > A	A = 0.0002	Pro142Leu	CPD2	n.d.
25.	rs181882698	Missense	C > T	T = 0.0002	Asp75Asn	TMD2	n.d.
26.	rs141141857	Missense	G > A	C = 0.0002	Pro238Ser	TMD6	n.d.
			G > C		Pro238Ala		
27.	rs111606529	Missense	G > A	A = 0.0002	Arg290Trp	CPD4	n.d.
28.	rs528014472	Nonsense	C > A	A = 0.0002	Gly9Ter	ECD1	n.d.
29.	rs141744602^*^	Missense	C > T	T = 0.000008	Gly165Glu	ECD3	Lost (HK1, SP, IC, hBD3) (58)
30.	rs372988289^*^	Missense	C > G	G = 0.000008	Asp184His	TMD5	Lost (HK1, SP, IC, hBD3) (58)

SNPs were extracted from NCBI dbSNP database (http://ncbi.nlm.nih.gov/SNP/) or Exome Aggregation Consortium (http://exac.broadinstitute.org/). SNPs validated by at least 1,000 Genomes Project are shown except SNPs marked with

* *. The localization of MRGPRX2 domains is based on work published by Reddy et al. (53). ECD, extracellular domain; CPD, cytoplasmic domain; TMD, transmembrane domain; n.d., not determined; SP, substance P; hBD3, human β-defensin-3; HK1, hemokinin-1; IC, icatibant*.

Basophiles are the blood cells corresponding to mucosal and tissue mast cells in respect of functional and structural features. Evidence on MRGPRX2 involvement in basophile activation is limited. Some recent pilot data showed that surface expression of MRGPRX2 on basophiles significantly increased upon unspecific stimulation, but data come from a single conference report (59) and should be treated as preliminary information.

In summary, the mice Mrgprb2 and the human ortholog MRGPRX2 are activated by basic secretagogues and neurokinins, but also a number of peptidergic drugs (i.g. icatibant) that frequently induce the injection-site reactions (Supplementary Figure S2) as well as small-molecule drugs (NMBA, fluoroquinolones, vancomycin) that may produce anaphylactic events. In addition, a drug-induced response is reduced in Mrgprb2 knockout mice. Main physiological actions and pathological relevance of the MRGPRX2 are summarized in Supplementary Table 1.

## Implications and Hypothesis

McNeil et al. demonstrated that distinct drugs elicit the MRGPRX2-related MC degranulation. The same drugs are also known to cause IgE-dependent MC degranulation, which results in producing local or systemic anaphylaxis (14). It has been shown with human and mice MCs cultures under laboratory conditions, but to what extent results can be translated into drug-hypersensitive patients remains the question.

Reactions are often observed upon first exposure in drug-naïve patients, almost always after injection with icatibant, but rarely during treatment with NMBA or fluoroquinolones. If the reactions share the MRGPRX2-dependent mechanism, why do they differ so much in respect of frequency and why do only a small minority of individuals in the general population develops NMBA-induced reactions? There are theoretical possibilities (i), that, even in the same subjects, some reactions are mediated by drug-specific IgE, the others by MRGPRX2; (ii) that positive skin tests reflect IgE-dependent sensitization and/or alternative degranulation pathway and both mechanisms may be responsible for cross-reactivity between drugs of interest.

One could also hypothesize that:

1. Mutation in the MRGPRX2 gene affects the risk of anaphylactic events being mediated by the MRGPRX2 receptor. Mutation in a single amino acid residue was shown to fail the activation of the receptor by the substance P and the compound 48/80 (53). In the same paper, the authors demonstrated that another receptor agonist, LL-37 activates both mutant and native receptor. The receptor antagonist QWF inhibits MCs activation with the substance P and the compound 48/80, but not with LL-37, as described above. Naturally occurring SNPs may abrogate MC-mediated degranulation in response to MRGPRX2 ligands including substance P, hemokinin-1, human β-defensin-3 and icatibant (58). Our analyses revealed several other SNPs that could potentially affect the ligand binding properties of MRGPRX2 (Table 1).
  Therefore:
2. Icatibant and NMBA may interact with the MRGPRX2 through different active-sites of the same receptor, which implies differences in frequency of induced reactions.
3. Also differences in intracellular molecular mechanisms underlying the signalosome of MRGPRX2 may implicate differences in response to stimulation.
4. Epigenetic modifications of MRGPRX2 due to environmental influences represent another possible source of differences in response to the drugs of interest. Although our own studies have not revealed the CpG islands within the promoter region of MGPRX2 gene (Supplementary Figure S3), methylation status even of a single CpG locus can modulate protein expression (60).
5. Post-transcriptional modification of RNAs, including capping, splicing, and polyadenylation, could potentially result in production of MRGPRX2 variants of different properties. However, until now only two transcript variants of MRGPRX2 have been described (43). Both encode the same protein. Novel RNA transcripts within different cells and tissues can be identified by RACE (rapid amplification of cDNA ends).
6. Alternatively, the expression of MRGPRX2 may vary between individuals or in a single individual, temporarily or constitutively, as it was shown in patients with chronic urticaria, who had a significantly higher number of MRGPRX2+ skin MCs and percentage of MRGPRX2+ MC among all MC in comparison to control subjects (46). One may speculate that some diseases such as cutaneous or/and systemic mastocytosis alter the physiological levels and response mediated by MRGPRX2 (34). Also, different MC phenotypes, as already aforementioned, differ in their shape, released mediators as well as their response to stimuli provoking degranulation (26). FcεRI-mediated mast cell activation involves an inflammatory response mediated by transcription factors (TFs) like AP-1, NF-kB, or NFAT (61). Currently, it is not known which TFs can regulate MRGPRX2 expression in response to different stimuli (Supplementary Figure S3).
7. A number of MC triggering agents have been described, e.g., drugs, food, temperature (37). It is likely that in some cases, merely the combination of co-factors has a cumulative effect strong enough to achieve a sufficient level of MC activation and to elicit degranulation. During anesthesia several co-factors may be present simultaneously (i.e., opioides, NMBA, temperature), which could explain why only certain patients react to NMBA. The incidence of NMBA-induced anaphylaxis was shown to be higher in the population exposed to pholcodine (a common over-the-counter antitussive). One explanation, widely discussed in published research on the subject, is the presence of IgE-dependent cross-reactivity between NMBA and pholcodine, as both possess similar ammonium structures (37, 62). On the other hand tertiary and quaternary ammonium (QA) structures are shared by many compounds, so possibly there is something more specific behind the relation between NMBA and pholcodine. Another explanation suggests that QA ions bind directly to immune receptors, including IgE on MC and cause their activation (62). The exact mechanism of this phenomenon remains unclear.
8. Further studies may uncover more MRGPRX2 ligands inasmuch as some other drugs, apart from those aforementioned, are known to induce non-IgE-dependent immediate reactions (e.g., iodinated contrast media). The MC degranulation in response to different triggering agents can exhibit distinct features and dynamics (33). One may hypothesize that rapid and transient change in drug concentration in local MCs milieu produces a stronger reaction than slower change. The simultaneous engagement of a larger number of MRGPRX2 on MC may be necessary to trigger degranulation. Such phenomenon would imply differences in hypersensitivity reactions between drugs given orally and parenterally, or provide additional insight into mechanisms of desensitization, an insight which is based on a re-administration of the drug with reduced speed.
9. Basophil activation tests are positive only in some of patients with immediate drug-induced reactions, for example with reactions due to exposure to moxifloxacin, belonging to fluoroquinolones (63). It is not known if a negative BAT reflects insufficient sensitivity of the method, or if a reaction is mediated by MRGPRX2 and is undetectable by means of measurement of CD63/CD203c expression in some cases. One may put forward the hypothesis that patients with the same phenotype share different or mixed endotypes of immediate reaction. Additional studies closing this gap in our knowledge would be of great value.
10. Taking into consideration the possible role of MRGPRX2 in drug-induced anaphylactic events, one of the burning questions is the following one: if there was a blockade of this receptor would we be able to prevent such episodes happening? Under laboratory conditions the tripeptide QWF was shown to inhibit MC degranulation induced, among others, by substance P, atracurium, and ciprofloxacin (52). Whether tyrosine-kinase inhibitors (masitinib) or MC stabilizers, such as sodium cromoglycate and ketotifen could block MRGPRX2-mediated reactions needs to be investigated (26, 64).

Addressing these issues would enable researchers to better understand mechanisms of immediate drug-induced reactions and may improve patient safety in the long term. Assuming that certain polymorphisms of the MRGPRX2 gene and expression levels of MRGPRX2 in the skin can pose a phenotype predisposing some individuals to immediate drug-induced reactions, a first possible approach of further studies could be as follows: prick and intradermal skin tests with drugs of interest (e.g., ciprofloxacin) performed with a very broad range of drug concentrations in a group of a sufficient number of healthy volunteers, who were exposed formerly to tested drugs and tolerated them. In this way, the individual threshold of response to a particular concentration of drug could be identified. In the literature the reported thresholds of non-irritant test concentrations of ciprofloxacin range from 0.000001 to 0.02 mg/mL (65). Then the correlation between the drug concentrations eliciting skin response and the expression of MRGPRX2 in the skin can be assessed, together with investigation of polymorphisms in the MRGPRX2 gene, which may affect receptor function. The latter one would be of interest, especially in the individuals presenting extreme responses in skin tests (responding to the highest and the lowest drug concentrations). In the next step the similar approach could be pursued to compare healthy controls and real patients with drug-induced reactions, who are obviously less available. The coexisting common IgE-depended drug allergy has always to be taken into account.

## Ethics Statement

This study was carried out in accordance with the recommendations of The First Local Ethical Committee on Animal Testing at the Jagiellonian University in Krakow. The protocol (41/2014) was approved by the First Local Ethical Committee on Animal Testing at the Jagiellonian University in Krakow.

## Author Contributions

GP and MK wrote the paper and analyzed data. KK carried out the experiments. MP helped design the figures. All the authors critically revised the manuscript.

### Conflict of Interest Statement

The authors declare that the research was conducted in the absence of any commercial or financial relationships that could be construed as a potential conflict of interest.
