# Supplementary material for: Mas-Related G Protein-Coupled Receptor-X2 (MRGPRX2) in Drug Hypersensitivity Reactions
Source: Front Immunol. 2018 Dec 20;9:3027. doi: 10.3389/fimmu.2018.03027 (PMC6306423; doi:10.3389/fimmu.2018.03027)
Supplement: Supplementary file 1 [file Data_Sheet_1.pdf]

Supplementary Material

Mas-Related G Protein-Coupled Receptor-X2 (MRGPRX2) in drug hypersensitivity reactions

Grzegorz Porebski[1], Kamila Kwiecien[2], Magdalena Pawica[1], Mateusz Kwitniewski[2*]

[1]Dept. of Clinical and Environmental Allergology, Jagiellonian University Medical College, Krakow, Poland

[2]Dept. of Immunology, Faculty of Biochemistry, Biophysics and Biotechnology, Jagiellonian University, Krakow, Poland

*** Correspondence:** Mateusz Kwitniewski (mateusz.kwitniewski@uj.edu.pl)

Supplementary Figure 1 - (A) Tissue distribution of human MRGPRX2 transcripts. The RNA-Seq data from the Human Protein Atlas projects (BioProject: PRJEB4337) were obtained from the Entrez Gene (http://www.ncbi.nlm.nih.gov/gene), National Center for Biotechnology Information (NCBI)'s database for gene-specific information (Gene ID: 117194). The data are shown as reads per kilobase of exon per million mapped sequence reads (RPKM) +/- standard deviation (SD), n >= 2 samples per tissue. (B) Electrophoretic analysis of MrgprB2 (murine ortholog of MRGPRX2) PCR products. Arrows indicate specific products, lower bands are primer-dimers. Preadipocytes were obtained from fat pads of 6-week C57BL6/J mice after collagenase D digestion and magnetic-activated cell sorting ($CD45^{-}CD31^{-}Sca\text{-}1^{+}$). Isolated preadipocytes were plated and cultured for 7 days, then differentiated for 3 days and maturated for another 7 days. Tissues were harvested from 8-week C57BL6/J mice. RNA was isolated from both cultured adipocytes and collected tissues. Afterwards, reverse-transcription PCR followed by quantitative real-time PCR were performed (MrgprB2: Fwd 5'CATTAGCATTGAGCGCTGCTT, Rev 5'CCTTCCAGGAGACCCAACAA, GAPDH: Fwd 5' TGTGTCCGTCGTGGATCTGA, Rev 5' TTGCTGTTGAAGTCGCAGGAG).

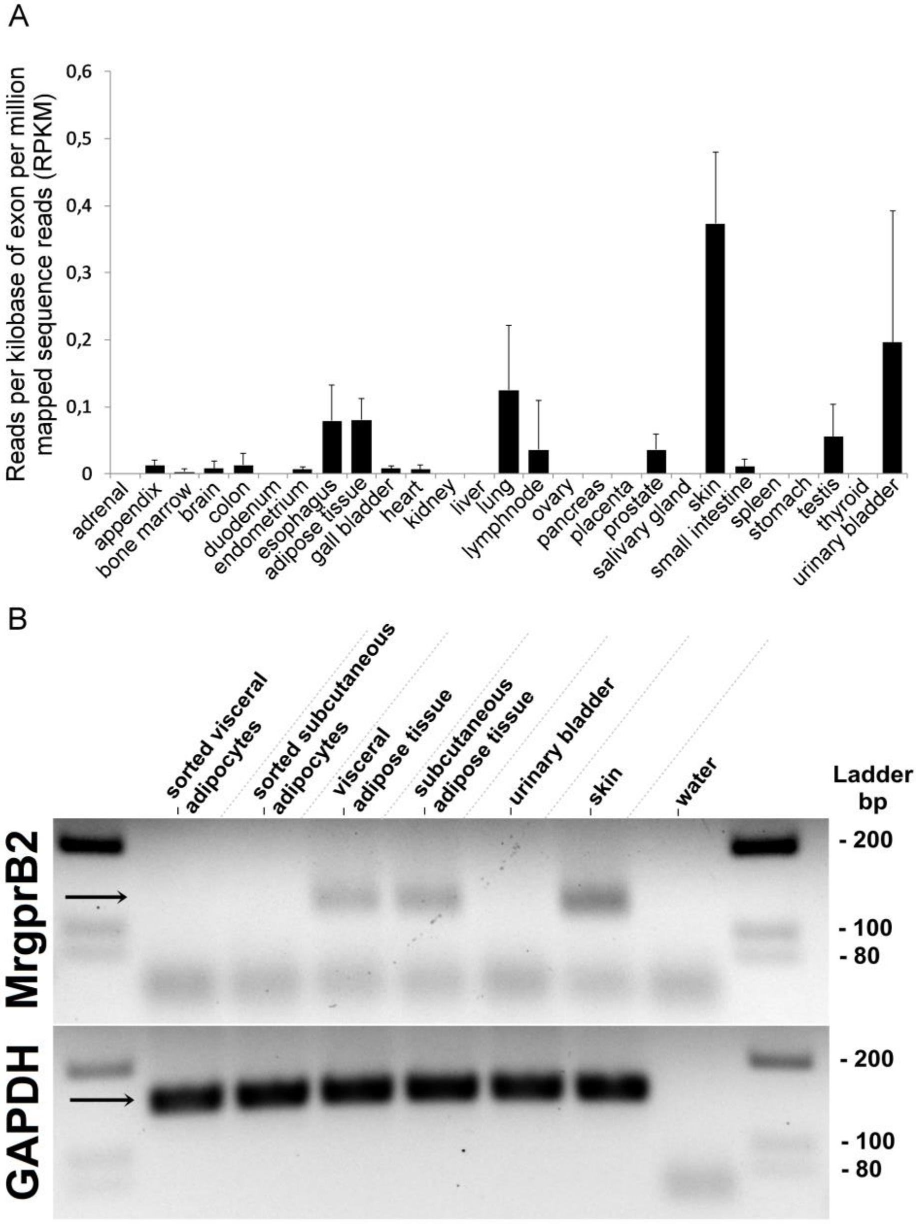

Supplementary Figure 2 - A local skin reaction induced by subcutaneous injection of icatibant (The written informed consent for the publication was given by the patient).

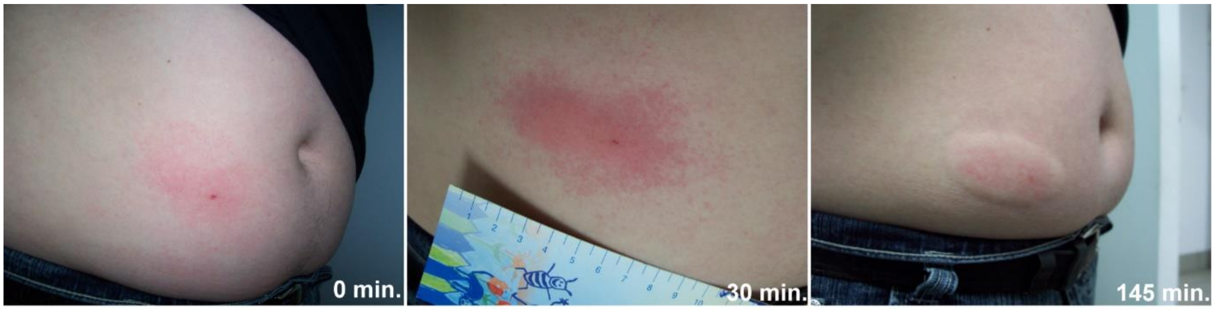

Supplementary Figure 3 - The location of the CpG sites in MGPRX2 gene within the first 1,000 bp upstream and downstream from the first transcription start site. The MRGPRX2 gene and promoter DNA sequence was downloaded from the NCBI Gene database (https://www.ncbi.nlm.nih.gov/gene/117194). The CpG sites were analyzed by EMBOSS Cpgplot online tool (https://www.ebi.ac.uk/Tools/seqstats/emboss_cpgplot/) with default settings (window: 100; minimum length of an island: 200 bp; minimum observed to expected ratio: 0.6; minimum average percentage of G plus C: 50) and online tool MethPrimer (http://www.urogene.org/cgi-bin/methprimer/methprimer.cgi) with default settings (window: 100; shift:1; Obx/Exp: 0.6; GC%: 50%). The CpG island were not detected within indicated DNA region. TF – transcription factor. Transcription factors that could regulate the transcription of MRGPRX2 gene have not been identified yet.

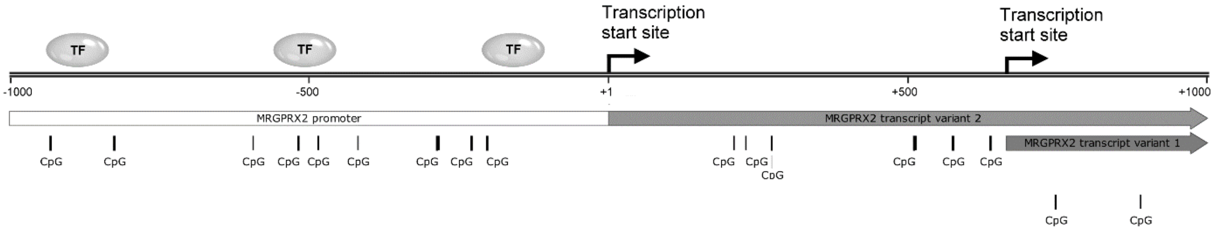

Supplementary Table 1 - Main roles of the MRGPRX2 in health and in diseases (14, 32, 38, 46, 54, 58).

Receptor agonists	Clinical relevance
Host defense peptides: β defensins and cathelicidins (LL-37)	Innate immunity and linkage to adaptive immunity, healing of wounds (release numerous of physiologically relevant mediators, which increase vascular permeability, attract leukocytes to the area of insults, promote migration of dendritic cells and indirectly enhance antigen presentation to T cells)
Small-molecule drugs: neuromuscular blocking agents (atracurium, cisatracurium, tubocurarine); fluoroquinolones (ciprofloxacin, moxifloxacin, levofloxacin, ofloxacin)	Systemic drug-induced hypersensitivity reactions (so called pseudo-allergic, or anaphylactoid reactions), including anaphylaxis
Cationic peptidergic drugs: icatibant, cetrorelix, leuprolide, sermorelin	Injection-site reactions (erythema and swelling)
Vancomycin	Red man syndrome (erythema and itch usually involving the face, neck and torso)
Neuropeptides (e.g. substance P, vasoactive intestinal peptide)	Neurogenic inflammation, which contributes to chronic urticaria and possibly to some cases of severe bronchial asthma
